# A Layered Control Architecture of Sleep and Arousal

**DOI:** 10.3389/fncom.2020.00008

**Published:** 2020-02-12

**Authors:** Michael C. Chen, Siamak K. Sorooshyari, Jian-Sheng Lin, Jun Lu

**Affiliations:** ^1^Department of Neurology, Beth Israel Deaconess Medical Center, Harvard Medical School, Boston, MA, United States; ^2^PureTech Health, Boston, MA, United States; ^3^Department of Integrative Biology, UC Berkeley, Berkeley, CA, United States; ^4^Centre de Recherche en Neurosciences de Lyon, Bron, France

**Keywords:** sleep-wake regulation, layered model, discrete event system (DES), ascending reticular activations system, hierarchical system

## Abstract

Sleep and wakefulness are promoted not by a single neural pathway but via wake or sleep-promoting nodes distributed across layers of the brain. We equate each layer with a brain region in proposing a layered subsumption model for arousal based on a computational architecture. Beyond the brainstem the layers include the diencephalon (hypothalamus, thalamus), basal ganglia, and cortex. In light of existing empirical evidence, we propose that each layer have sleep and wake computations driven by similar high-level architecture and processing units. Specifically, an interconnected wake-promoting system is suggested as driving arousal in each brain layer with the processing converging to produce the features of wakefulness. In contrast, sleep-promoting GABAergic neurons largely project to and inhibit wake-promoting neurons. We propose a general pattern of caudal wake-promoting and sleep-promoting neurons having a strong effect on overall behavior. However, while rostral brain layers have less influence on sleep and wake, through descending projections, they can subsume the activity of caudal brain layers to promote arousal. The two models presented in this work will suggest computations for the layering and hierarchy. Through dynamic system theory several hypotheses are introduced for the interaction of controllers and systems that correspond to the different populations of neurons at each layer. The models will be drawn-upon to discuss future experiments to elucidate the structure of the hierarchy that exists among the sleep-arousal architecture.

## Introduction

The complex and varied behaviors of animals in their environment require the general arousal of the nervous system. While awake, animals can process information from the environment and generate a diverse set of behaviors. During sleep, both the responsiveness to the environment and the complexity of behavior is greatly reduced. Sleep and wakefulness are often measured with the ensemble activity of neurons of the mammalian cortex using electroencephalography (EEG), along with motor activity as measured by muscle electromyography (EMG). High-frequency, asynchronous, “activated” EEG is thought to be a marker for the information flow that underlies consciousness (Koch et al., 2016). In contrast, coma patients have an oscillating, monotonous, and uninformative EEG (Posner et al., 2007). Sleep is not simply the absence of behavior since it involves coordinated neuronal activity. Sleep and wake are complex, actively regulated states, and both are ultimately synchronized on the level of the organism. How these behavioral states are initiated, coordinated, and regulated is a crucial question in neuroscience.

One method of understanding sleep and wake is to consider how the structure and function of the mammalian brain serves to implement an algorithm to arrive at a behavioral state (Krakauer et al., 2017). Such algorithmic approaches are not new; for example, a directional flow of neuronal activation, referred to as the ascending reticular activating system, has been a prevalent neuroanatomic model for sleep-wake regulation (Moruzzi and Magoun, 1949). This model has helped frame the connections between various sleep and wake regulating nuclei in the brain (Edlow et al., 2012). We present a novel model for understanding the interaction of brain regions in producing sleep and wake states. Supporting empirical evidence is reviewed and we predict features of sleep and arousal-regulating circuitry based on the model.

## Layered Control

The interactions between discrete nuclei in the neuraxis coordinate behaviors such as arousal and sleep. Historically, in “ascending” models of arousal, information from basal neural structures in the brainstem flows rostrally to structures in the forebrain. Separating the rest of the brain from the brainstem (midbrain transection) or severe damage to the brainstem results in a coma (Bremer, 1935), while electrically stimulating certain brainstem structures (pontine reticular formation) results in wake-like EEG activity (Moruzzi and Magoun, 1949). The principles of ascending arousal have led to novel experiments integrating neuroanatomy, neurophysiology, and behavior (Munk et al., 1996) and continues to guide interpretation of experimental data and visualization of the interactions between nuclei (Brown et al., 2012). However, the neuroanatomy of the ascending reticular activating system (ARAS) has been reconsidered, showing that canonical “wake-promoting” nuclei or brain regions have a limited gross effect on arousal, while specific nodes such as the parabrachial nucleus (PB) play a crucial role in arousal (Fuller et al., 2011). Sleep-promoting nuclei, on the other hand, have been found throughout the neuraxis (Gerashchenko et al., 2008; Anaclet et al., 2012, 2014; Qiu et al., 2014; Xu et al., 2015). Models of sleep and wake, including models for rapid-eye movement (REM) sleep (Lu et al., 2006) and sleep transitions (Sorooshyari et al., 2015) have started to incorporate such findings.

**Figure 1:**
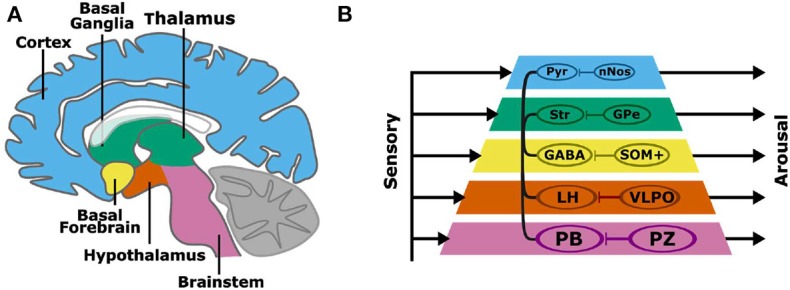
A subsumption architecture for layered control of arousal **(A,B)**. Colored layers of the neuraxis contain both wake promoting and sleep-promoting neurons. Layers have separate sensory inputs (left) and arousal behavior outputs (right). The combined outputs result in the complex behavioral profiles of sleep and wakefulness. Wake-promoting neurons project widely throughout the neuraxis, while sleep-promoting neurons project locally and target wake-promoting neurons within each neuraxis layer. Basal layers have a stronger behavioral output, and layers that are higher in the pyramid can subsume lower layers to promote arousal. The connections within the pyramid indicate the interaction among layers in the hierarchy, namely brain regions containing wake-promoting neurons. A more specific instantiation of the subsumption architecture is shown involving wake-promoting parabrachial nucleus (PB), lateral hypothalamus (LH), GABAergic basal forebrain (BF) neurons (GABA), striatum (Str), and cortical pyramidal (Pyr) neurons, and sleep-promoting parafacial zone (PZ), ventrolateral preoptic nucleus (VLPO), somatostatin-positive (SOM+) neurons, external globus pallidus (GPe), and cortical neuronal nitric oxide synthase (nNOS) expressing neurons.

Non-linear systems can produce complex behaviors with simple rules and structures. For example, the behavior of a robot can be generated from simple and interacting functional modules. One approach, the subsumption architecture proposed by Brooks, is to layer modules, each with independent inputs and outputs (Brooks, 1986). In this layered control architecture, complex behaviors can emerge without a consolidated output pathway. Higher layers can subsume the commands of lower layers, and the sum of these parallel processes is a robust behavioral output. The organization of the layered control architecture modules resembles the layered evolutionary expansion of the brain (Prescott et al., 1999). Beyond the brainstem, layers of circuits have been added to the architecture of the vertebrate forebrain, including the diencephalon (hypothalamus, thalamus), basal ganglia, and cortex (Figure 1A). The aforementioned structures have distinct genetic, morphological, and functional identities. Layered control architecture can be applied to the modules within the brain to represent how disparate nuclei interact to regulate complex wake or sleep behavior.

We propose a model of sleep-wake regulation, based on a layered control architecture (Figure 1B). The presented model suggests that arousal is maintained by parallel wake-promoting circuits within multiple layers with each layer equated to a brain region. Rather than a single center or converging pathway for arousal or sleep, complex behaviors including sleep and arousal arise from the combined outputs of multiple processing layers. The model in Figure 1 has several key features:

- Regions of the brain, and the wake-promoting or sleep-promoting neurons within the regions/layers, have independent inputs and outputs.
- The caudal (e.g., brainstem) wake- and sleep-active neuronal groups have the strongest overall control of sleep and wakefulness.
- Wake-active neurons can project to and communicate with other layers of the brain to consolidate states of arousal.
- Sleep-promoting neuronal groups act locally by inhibiting nearby wake-active centers to promote a global brain activity state.
- Rostral layers (such as cortex), despite less overall sleep-wake control, can subsume lower layers to regulate overall sleep-wake behavior.

It should be noted that in referring to wake- and sleep-promoting neural populations, we consider both nuclei and sub-populations. In several cases there is sufficient resolution within a nuclei so that a definitive statement can be made about a sub-population of neurons, and in other scenarios such resolution does not exist and it will be necessary to make a collective statement about whether that nuclei/brain region is implicated more so in promoting arousal or in promoting sleep.

## Networked Control of Wakefulness

Although sleep and wakefulness are often measured via cortical activity, the cortex itself is not necessary for either behavior. Removal of the cortex, or decortication, does not eliminate wakefulness in dogs (Kleitman and Camille, 1932), rats (Vanderwolf et al., 1978), cats (Villablanca, 1966), or mice (Wenzel and Lal, 1959). Similarly, after transection at the midbrain, cycles of activity and rest reminiscent of sleep remain, after recovering from coma like state. However, a decerebrate animal may show reduced or disorganized behavior due to the reduction in arousal and also from the destruction of the brain circuitry necessary for organizing other behaviors (Chen et al., 2016).

Wake-active neurons increase firing during wake and contain neurotransmitters such as glutamate, orexin, dopamine, and acetylcholine (Brown et al., 2012). Notably, each layer of the brain contains wake-promoting neurons. In the brainstem, there are numerous wake-promoting nuclei, including the PB, laterodorsal pontine tegmentum (LDT), pontine pedunculopontine tegmentum (PPT), and locus coeruleus (LC). The basal forebrain (BF) itself is thought to contain wake-promoting sub-populations, most likely GABA producing neurons (Xu et al., 2015), although the basal forebrain has a much stronger function in the regulation of cortical activity (Kim et al., 2015) or possibly sleep-wake transitions (Han et al., 2014) than driving overall wakefulness. Similarly, basal ganglia neurons regulate cortical activity and can promote wakefulness (Qiu et al., 2010).

Due to redundancy in wake-promoting circuitry, specific nodes can specialize to support facets of arousal at various behavioral situations. For example, dopaminergic dorsal raphe neurons (DRN) project to the amygdala and drive motivated behavior to salient stimuli (Cho et al., 2017). Individual arousal systems, like the histaminergic tuberomammillary nucleus, may be less critical for sustaining overall arousal (Gerashchenko et al., 2004) while playing a role in the overall wake network. Other nodes may contribute to specific facets of arousal, such as the role of orexinergic neurons in synchronizing and stabilizing the overall wake state, or the role of BF and LDT/PPT cholinergic neurons in regulating cortical synchronization (Adamantidis et al., 2007; Boucetta et al., 2014; Anaclet et al., 2018).

The wake-active neurons are widely distributed in the brainstem and forebrain, and interconnect to form a wide-projecting network. For instance, the wake-active PB in the brainstem projects to the wake-active lateral hypothalamus (LH), basal forebrain, thalamus, and cortex. The LH projects to the wake-active BF, which in turn projects to the cortex, as well as to the brainstem PB, resulting in concerted wake-active activity in numerous wake-promoting systems. This connectivity, in addition to the aforementioned functional redundancy, indicates that some wake-promoting systems, such as the LC or DRN, can be damaged without compromising an animal's overall behavioral state. However, damage to the caudal wake nodes—the PB in particular—leads to hypersomnolence and a coma-like state (Fuller et al., 2011).

A key feature of the layered control architecture is the ability of rostral structures to subsume caudal wake-promoting nodes. The cortex can, through descending projections, promote arousal to enhance and stabilize the sleep-wake states (Aston-Jones and Cohen, 2005; Cano et al., 2008). This subsumption enables voluntary wakefulness but also may play a role in sleep disorders such as insomnia where aberrant wake-like cortical activity is sometimes observed alongside difficulty falling asleep (Bonnet and Arand, 2010). Increasing cortical mass may allow increasing subsumption of caudal wake-promoting structures and reduce sleep. For example, increasing brain size with body mass in mammals is correlated with decreasing the need for sleep (Herculano-Houzel, 2015). One prediction from this model would be that expanded brain size can also stabilize sleep or wake states, resulting in longer bout durations, a trend that is observed across several mammalian species (Lo et al., 2004).

## Local Control of Sleep Promoting Systems

Like wakefulness, sleep is a globally coordinated state although it can also be regulated locally (Krueger et al., 2008; Vyazovskiy et al., 2011). In contrast to wake-active neurons, non-REM sleep (NREM) or NREM-rapid eye movement sleep (REM) active neurons contain inhibitory neurotransmitters such as GABA, glycine, and galanin. So far, NREM and NREM-REM sleep-active neurons have been identified in the parafacial zone (PZ) in the brainstem, ventrolateral preoptic nucleus (VLPO) and median preoptic nucleus, melanin-concentrating hormone containing neurons, a subpopulation of GABAergic neurons in the basal forebrain, and Nos (GABA) interneurons in the cerebral cortex.

Unless many more undiscovered sleep-promoting regions exist, it is likely that there are more wake-promoting loci than sleep-promoting loci. Unlike the wake-promoting systems, the existing sleep-promoting centers project to and act on wake-active neurons, with the strongest enervation within a layer (e.g., brainstem projections within brainstem), although wider and sparser projections exist. For example, the sleep-active PZ projects to the wake-active PB (Anaclet et al., 2012), and Nos (GABA releasing) neurons inhibit wake-active pyramidal neurons in the cerebral cortex (Gerashchenko et al., 2008; Morairty et al., 2013). While more in-depth functional neuroanatomic circuit mapping is needed, sleep-active populations of neurons from different layers do not appear to connect directly to each other. That is, the PZ does not appear to directly innervate other sleep-active neurons such as those within the VLPO, median preoptic area, or BF. In summary, sleep nodes, unlike wake nodes, are not networked together.

Based on the identities and physiological profiles of the known sleep nodes (Luppi et al., 2017), the most likely function of sleep-promoting nodes is inhibition of local wake-promoting neurons that in-turn inhibit the sleep-promoting neurons (Anaclet et al., 2014). This local control of sleep suggests that caudal sleep-promoting neurons play a stronger role in sleep promotion because they target stronger wake-promoting neurons. Indeed, brainstem PZ and hypothalamic VLPO have a crucial impact on sleep (Lu et al., 2000; Anaclet et al., 2012), while cortical Nos cells may have a weaker overall sleep-promoting output (Morairty et al., 2013). A second implication is that this local sleep-promoting control is, as a whole, less effective for producing a coordinated output (e.g., sleep) than wake-promoting systems that are networked and mutually reinforcing. Indeed, repeatedly activating the brainstem PB extends wakefulness despite high sleep pressure and activation of sleep-active neurons (Qiu et al., 2016). If sleep is a local phenomenon, an unanswered question is whether there is a single population of neurons that homeostatically regulates sleep need across the entire brain or if separate but interacting homestats exist throughout the brain but interacting to produce sleep behavior. While the layered control architecture proposed here does not provide an answer to this question, it does provide a framework for linking potential drivers of homeostatic sleep, like metabolite clearance (Xie et al., 2013), with a mechanism for implementing cell-level commands on a circuit or organ level.

## Coordinated Output of Sleep and Wakefulness

Ascending models of arousal have terminal targets, typically in the cortex, to produce a singular behavioral output. In the layered control architecture, each layer produces a separate output that is integrated at the level of the organism. For producing consolidated sleep and wake behaviors, the relationship between sleep-active and wake-active neurons is reflected in the firing activity patterns during sleep, wake, and the transition states. Arousal neuron firing increases prior to wake onset and decreases prior to sleep onset. Sleep-active neurons, on the other hand, lag in their response in firing—this suggest that the arousal neurons act first followed by sleep-active neurons (Takahashi et al., 2009; Sakai, 2011, 2014).

The relationship between sleep-active and wake-active neural populations suggests that arousal systems not only drive wakefulness but also permit sleep by deactivating arousal systems. Sleep systems, by suppressing arousal systems, elongate sleep after initiation. Strong sensory stimulation promotes arousal, while lack of sensory inputs may combine with inputs from sleep-active neurons to promote sleep. The link between regulatory elements and arousal is also reflected in the neuroanatomic overlap between sensory inputs and wake-promoting circuits. For example, the PB receives spinal cord sensory and gut visceral inputs as well as auditory and visual inputs, whereas none of the known sleep-active nodes receive direct sensory inputs.

It is likely that homeostatic forces act simultaneously and independently on both sleep and wake systems. However, arousal still can be generated even if sleep-active neurons are active (Qiu et al., 2016), supporting the relative dominance of wake-promoting systems. Similarly, we hypothesize that circadian control from the suprachiasmatic nucleus targets the arousal system. For example, the suprachiasmatic nucleus projects to the ventral sub-paraventricular zone (Lu et al., 2001), which then projects to dorsomedial and posterolateral hypothalamic arousal systems (Chou et al., 2003). Interestingly, some of these projections may relay in putative sleep-promoting regions (Luo and Aston-Jones, 2009). On the other hand, sleep-active neurons are mostly regulated by inputs of arousal systems and sleep pressure. Even local sleep pressure, homeostatic or circadian, is likely to have a stronger impact on more caudal, critical wake-promoting nodes (e.g., PB and posterolateral hypothalamus) directly, rather than an indirect effect on the sleep-promoting nodes that inhibit and are also inhibited by their partner wake-promoting nodes.

REM sleep (also known as paradoxical sleep) is a unique state that has both sleep features, such as muscle atonia, and wake features, such as cortical activation. REM sleep is driven by glutamatergic REM-active neurons that project to and drive the arousal network to recapitulate wake-like activity in the brain (Saper and Fuller, 2017). Specific REM relay neurons in the arousal network are both wake and REM-active (Boucetta et al., 2014). Concurrently, REM-sleep generator neurons have a unique descending pathway for muscle atonia control (Arrigoni et al., 2016). The REM sleep generator is under direct inhibitory control of GABAergic neurons in the ventrolateral periaqueductal gray and lateral pontine tegmentum (Saper and Fuller, 2017). These GABAergic neurons are regulated in turn by a variety of inputs including the VLPO, pedunculopontine laterodorsal tegmentum, LC, dorsal raphe nucleus, and medial prefrontal cortex (Saper and Fuller, 2017). REM sleep is a behavioral state influenced by both sleep and wake systems and can be modeled directly using a layered control architecture.

## Modeling Considerations

Two models are provided that incorporate dynamic system theory into the anatomical and functional views of Figure 1. We emphasize layering to demystify the complexity that is present in a network created by nature. In a layered architecture, the operation of individual circuits, i.e., the nodes, can be isolated for electrophysiological modeling via techniques such as dynamic causal modeling (DCM). The first model that we discuss follows directly from the subsumption architecture proposed by Brooks. Specifically, we suggest several computations performed by the neural circuits in the network's layers and discuss how these computations modulate the firing rates leading to sleep or arousal. The second model, a discrete event system (DES), represents the interactions and dynamic signaling that occurs between nodes in the arousal system. While the two models draw upon disparate theory, they share similarities by being layered, hierarchical approaches that reflect coupling and feedback between components within a networked system.

### A Subsumption Model of the Arousal System

**Figure 2:**
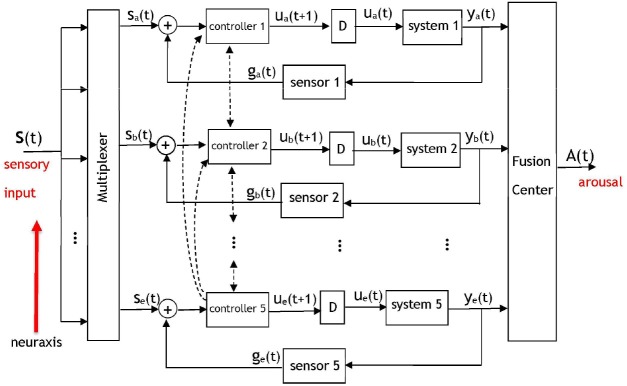
A dynamic system model of the pyramidal structure governing the arousal system. The five control loops correspond to five layers of neural circuitry discussed in Figure 1 that will contribute signals to a fusion center. Based on Brooks' subsumption model, inter-level (i.e., along the neuraxis) and intra-level hierarchy will exist among the components with respect to the potency of their signals at the fusion center. Each controller corresponds to a set of wake-promoting neurons at the respective layer, each system represents the sleep-promoting neurons at a layer, and “D” represents a temporal delay. A multiplexer determines the portions of the sensory input that is routed to each of the layers. The output of the fusion center will be an arousal signal denoting a behavioral state. The dashed lines indicate the presence of interaction between adjacent and non-adjacent controllers in the pyramidal hierarchy—the controllers of each layer communicate with controllers at other layers. The inputs to the fusion center are neural signals that have accumulated at different brain regions and amass to result in an output A(t) that represents an arousal state.

Brooks' comprehensive subsumption architecture has been used and expanded upon in multiple disciplines, including neuroscience. We suggest an implementation of Brooks' layered control architecture within the brain during governance of sleep and arousal. From an algorithmic perspective, the model shown in Figure 2 is comprised of a series of five communicating dynamic systems and serves as a system-theoretic description of Figure 1. We shall propose several computations as reasonable candidates for the processing performed by this model's components.

A salient aspect of the arousal system is the fact that its pyramidal structure facilitates direct and indirect communication among the layers; the activity of the hierarchically superior layers drives the activity of the less-potent layers. Although Brooks' work suggests that hierarchically superior layers may subsume the commands of less-potent layers, a certain degree of interaction takes place among the adjacent layers since they are anatomically connected. There will also be interaction between non-adjacent layers in the hierarchy, representing long-range anatomic projections. The hierarchical dynamic system in Figure 2 incorporates five control systems (Åström and Murray, 2008) that operate on disparate time-scales and on occasion receive no input from a multiplexer that routes different portions of the sensory input to the various layers. Anatomically, separate sensory pathways exist to the five layers. Peripheral or internal sensations have more direct inputs into the brainstem and thalamus than into the basal ganglia (Hylden et al., 1989; Herbert and Saper, 1990). The notion of a multiplexer serves as an abstraction for the neural mechanisms that govern the distribution of sensory information to the layers depicted in Figures 1, 2. Such neural mechanisms encompass systems of interneurons that route the activity of sensory neurons to the appropriate layer of the pyramid. In Figure 2 the five controllers correspond to the wake-promoting neurons at each respective layer, and the five systems represent the sleep-promoting neurons with a multiplexer gating the neural signal that is appropriate for processing at each layer. The notion of a fusion center has been drawn upon extensively in signal processing, communication theory, and control systems. A fusion center receives diverse, heterogeneous, processed information regarding a process and integrates the information to yield an aggregate signal denoting a decision (Thomopoulos, 1990). Although illustrated as a single unit, the fusion center is a distributed entity with components throughout the brain. The inputs to the fusion center are neural signals that have accumulated at different brain regions and amass to result in an output A(t) that represents an arousal state. This matches anatomical evidence against the existence of a single location where arousal-related activity is summed from heterogeneous circuits, but rather distributed nodes whose neural activity correlates with an organism's immediate arousal state (Saper and Fuller, 2017).

The dashed lines in Figure 2 denote the wake-promoting neurons' extensive projections to wake-promoting neural populations at other layers. Similar connections are not shown for the sleep-promoting neurons because they do not project as widely as wake-promoting neurons (Su et al., 2018). Rather, the sleep-promoting neurons primarily project locally within their respective layer—this is illustrated in the model via a delayed feedback path to reflect the influence of the sleep-promoting populations within their own layer. The feedback signaling is gated by a sensor that represents a conductance to the transmission of downstream neural activity to upstream sources. A sensor network within the context of a biological system is viewed as a set of entities (e.g., neural circuits) that monitor, record, and relay the physical conditions of the environment to another entity (e.g., a controller). The intra-layer delay (denoted by “D”) between the response of the sleep-promoting and wake-promoting neurons has been discussed in works such as Takahashi et al. (2009) via single-unit recordings from a large number of neurons that spanned several brain regions. Although the delay values are important in the operation of the model and affect the system dynamics, the model is not contingent on specific delay values. From an algorithmic perspective the sensory signals input to the multiplexer in Figure 2 are represented as a vector *s*(*t*) = [*s*_1_(*t*), *s*_2_(*t*), *s*_3_(*t*), *s*_4_(*t*), *s*_5_(*t*)] with components that are permuted to the appropriate control system by the multiplexer to form the input set {*s*_*a*_(*t*), *s*_*b*_(*t*), *s*_*c*_(*t*), *s*_*d*_(*t*), *s*_*e*_(*t*)}. Without loss of generality we shall perform the analysis on the uppermost layer indexed with subscript “a.” The dynamics for a control loop in the architecture are proposed as

(1) $$\begin{array}{l} u_{a}\left(t+1\right)=C_{1}\left(s_{a}\left(t\right)+g_{a}\left(t\right)\right)\\ y_{a}\left(t\right)=u_{a}\left(t\right)*h_{1}\left(t\right)\\ g_{a}\left(t\right)=S_{1}\left(y_{a}\left(t\right)\right) \end{array}$$

where C_1_(.) denotes the control rule, or synonymously, the function applied to the aggregate neural activity s_a_(t) + g_a_(t) by a controller. The impulse response of the system is given by h_1_(t), ⋇ denotes the convolution operator, and the intermediate variable u_a_(t) represents the control signal that is applied to a system. The sensor S_1_(.) models a conduit of interaction between a system and controller. The scenario of *g*_*a*_(*t*) = *S*_1_(*y*_*a*_(*t*)) = *y*_*a*_(*t*) denotes an untransformed and unperturbed communication of information from the sleep-promoting population to the wake-promoting population. Conversely, the case of *g*_*a*_(*t*) = *S*_1_(*y*_*a*_(*t*)) = 0 represents an ablation in the feedback connection. Naturally, neither the unperturbed nor ablated scenarios are expected to be representative of normal, healthy function—rather S_1_(.) is hypothesized to be a non-linear function that contains stochastic noise. The signal output by the fusion center in Figure 2 is then described by

(2) $$\begin{array}{r} A\left(t\right)=f_{1}y_{a}\left(t\right)+f_{2}y_{b}\left(t\right)+\;\ldots \;+f_{5}y_{e}\left(t\right) \end{array}$$

with *f*_*i*_:*i* = 1, 2, …, 5 representing adaptive weights that take values on a continuous interval from 0 to 1 and determine the relative importance of the fusion center's signals during the formation of A(t). The weights that comprise the summation at the fusion center are also dynamic processes with values that may change on a disparate time-scale. This is because the fusion center is comprised of the summation of responses of the neural circuits with a response—modulated via the weights— which is also brain-state dependent. Obviously, a value of *f*_*i*_ = 0 indicates that the *i*th layer will not actively contribute to the procurement of the behavioral state as quantified by the signal A(t). It should be apparent that the terms comprising the summed components of the fusion center are the outputs of the neural circuits of the layers in Figure 1, namely from the wake-promoting PB, LH, GABA basal forebrain, striatum, and cortical pyramidal neurons, and the sleep-promoting PZ, VLPO, somatostatin-positive (SOM+) basal forebrain (Xu et al., 2015), external globus pallidus (GPe), and cortical neuronal nitric oxide synthase (nNOS) expressing neurons.

Equations (1) and (2) represent a non-unique implementation of the system described in Figure 2. It is possible for the model to be altered or further refined as new data becomes available that ascertains the functional roles of the five layers. In the meantime, extant data allows for propositions to the functions in Equation (1). For instance, a possibility for the controller C_i_(.) is a sigmoidal operation

(3) $$\begin{array}{r} C_{i}\left(x\left(t\right)\right)=\frac{\alpha_{i}}{2}\left[1+\text{erf}\left(\frac{\;x\left(t\right)-\beta_{i}}{\sqrt{2\gamma_{i}}}\right)\right] \end{array}$$

where α_i_, β_i_, and γ_i_ are free parameters and erf(.) denotes the error function integral. If the signals in Equation (1) were firing rates, a sigmoidal form for C_i_(.) would be justified from a Wilson-Cowan model of firing rates for neuronal populations (Booth and Diniz Behn, 2014). The aggregate filtering or smoothing operation applied by the sleep-promoting neurons to the activity of the wake-promoting neurons would be modeled via h_i_(.) in Equation (1) being a Gaussian function. Such a choice captures the smoothing of a control signal u(t) that may have discontinuities. Obviously, the Gaussian will be parameterized by a (possibly time-varying) mean and variance whose values will need to be determined via a parameter estimation technique applied to the data. The sensor may be modeled by $S_{i}\left(y\left(t\right)\right)={y\left(t\right)e}^{-\lambda_{i}t}$ where a constant λ_*i*_ > 0 accounts for a decay in the importance of temporally distant neural activity.

Two types of hierarchy are present in the model: inter-level hierarchy between the layers, and intra-level hierarchy among the wake- and sleep-promoting neurons at a layer. Intra-layer hierarchy has been discussed already via a controller having a greater influence than a system on the input to the fusion center. With respect to inter-level hierarchy, the neural circuits situated lower in the neuroaxis are the most potent in their capability to induce arousal. Figure 1 illustrates the brainstem (more precisely, the PB) as being the most potent unit in contributing to arousal, followed by the lateral hypothalamus, and the cortex having the least influence among the five considered units. The presented model will account for this via the assignment C_5_(.) > C_4_(.) >… > C_1_(.) designating a hierarchy in controller outputs that contribute to the induction of an arousal state. As a result of the controller hierarchy, the relationship y_e_(t) > y_d_(t) >… > y_a_(t) is expected as the neural signals converge at the fusion center.

Ablation of any of the five controllers in the layered control architecture will influence an input to the fusion center and effect the arousal signal A(t). A subtler phenomenon is the effect that the ablation of a system or sensor will have upon the other units. Initially, all components within the network may not recognize that the ablation has occurred and continue to project activity to the ablated/malfunctioning unit as if it were in-tact. However, we hypothesize that the coupled nature of the system will eventually lead to the multiplexer being informed of an anomalous downstream target and, via synaptic plasticity, adapting its routing to attempt to reach an equilibrium. An equally important notion that this model may address is the prospective adaptation of the controllers at other layers to compensate for the lesions. As an example, consider the ablation of any one controller in Figure 2. While it is disputable to what degree the four remaining controllers would be affected, it is expected that the hierarchy would have a profound role in answering this question.

### A Discrete Event System Model of the Arousal System

**Figure 3:**
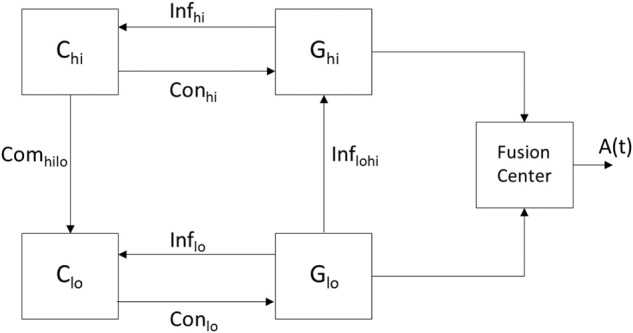
A modified version of the system in Zhong and Wonham (1990) illustrating hierarchical control of a DES and encompassing the unison of the states attained in G_lo_ and G_hi_ as contributing to the aggregate result. The resultant signal A(t) represents an arousal state such as wake or sleep with the contributions to the fusion center being the output of the neural circuits of various brain regions. Since high-level control by C_hi_ can be realized through the implementation of the lower level components (lo), the DES is an appropriate model for the pyramidal structure governing the arousal system. The hierarchy is in-place but the interactions among the component must be further specified as they will determine whether the populations of wake and sleep promoting neurons exceed/not-exceed thresholds associated with wake and sleep, respectively.

A DES is a dynamic system with a set of discrete states and transitions among the states—referred to as events—that occur at possibly unknown, irregular intervals (Ramadge and Wonham, 1989). A rationale for modeling the arousal system via a DES lies in the fact that the behavioral states comprise a finite set (i.e., REM, NREM, and wake) with the transition between the states being uncertain and prone to perturbation. The neural circuits participating in the communications that lead to a behavioral state may be viewed as a finite number of entities each containing a large number of constituent neurons and synapses. The collective work of Wonham, Zhong, and colleagues has been largely based on the architecture shown in Figure 3 (the authors did not consider a fusion center) and used in the hierarchical control of a DES (Zhong and Wonham, 1990). Their work has provided a theoretical basis to model the coupling among high-level and low-level control as well as the information channels that come into play in achieving such a unison. More specifically, in Figure 3 the communicating low-level (lo) and high-level (hi) architectures are each comprised of a system (G) and a controller (C). It is each controller's objective to drive a particular response in its associated system by providing commands on its control (Con) channel while receiving feedback from its efferent system via an information (Inf) channel. A pragmatic point in the work by the aforementioned authors is how to define the low- and high-level systems, G_lo_ and G_hi_, so that a high level controller C_hi_ can make effective control decisions to derive desired responses at both G_lo_ and G_hi_. In light of the hierarchical control that exists among the sleep-promoting and wake-promoting neural populations of the arousal system, an objective of this work is to suggest models that encompass the computations. We describe how a DES can model the operation of a regulatory system that encompasses an interaction among neural circuits to yield an arousal state. The components in Figure 3 are instantiated within the context of the arousal system by having the controllers C_lo_ and C_hi_ represent neural circuitry associated with sleep- and wake-promoting neurons, respectively. Furthermore, the systems G_lo_ and G_hi_ denote evolving behavior that is affected by the controllers as well as history and exogenous (i.e., environmental) factors. Just as there exists a hierarchy between the potency of the wake-promoting and sleep-promoting neurons, Figure 3 was derived with the high-level system having hierarchy over the low-level system. The “manager” C_hi_ is attempting to drive its system G_hi_ to a specific state and provides commands to C_lo_–the “operator”—to drive a response in the system G_lo_. Owing to the hierarchy between the high- and low-level systems, the induced arousal state in the high-level system may override that of the low-level system.

Zhong and Wonham consider a five-tuple of variables to study a two-level controlled discrete-event system (Zhong and Wonham, 1990). Their work advances prior DES theory by presenting the notion of hierarchical consistency. We consider a simplified and less rigorous version of the DES model that consists of the low-level system being described by a four-tuple

(4) $$\begin{array}{r} G_{lo}=\left(\Sigma_{lo},\;Q_{lo},\;\delta_{lo},\;q_{lo,0}\right) \end{array}$$

with Σ denoting the set of possible events, Q representing the set of states, q_0_ the initial state, and δ denoting a function that maps the combination of events and prior states into a subsequent state i.e., δ:Σ × *Q* → *Q*. For the low-level portion of the arousal system we consider

(5) $$\begin{array}{l} \Sigma_{lo}=\left\{s_{1}^{+},s_{1}^{-},\;s_{2}^{+},\;s_{2}^{-},\;\ldots ,\;s_{5}^{+},\;s_{5}^{-}\right\}\\ Q_{lo}=\left\{{\text{REM}}_{1},\;{\text{REM}}_{2},\ldots ,{\text{REM}}_{\text{K}},{\text{NREM}}_{1},{\text{NREM}}_{2},\ldots ,{\text{NREM}}_{\text{K}}\right\} \end{array}$$

where the states are the stages of sleep and the decreasing subscript values denote a higher valence toward the behavioral state. The possible events in Σ_*lo*_ represent the activity of the sleep-promoting neurons of the five layers in Figure 1 either exceeding (+ superscript) or not-exceeding (– superscript) a threshold associated with sleep (s). Qualitatively, a state of REM_1_ will denote the deepest state of REM sleep attained by the G_lo_ system. Specification and consideration of an initial state q_0_ is not trivial. Within a system-theoretic setting the initial conditions are usually specified or determined from the constructed system. Obviously, it is infeasible and perhaps futile to attempt to arrive at an arousal system's first ever bouts of sleep. Rather, the initial condition should be specified as a reference far enough back in time to account for the present arousal state. Thus, we posit that a choice of q_0_ is not unique. The transition function δ is unknown and its properties need to be discovered via experiments and subsequent data analysis. It is sensible to consider δ as a thresholding operation such as a sigmoidal function. Since the low-level controller *C*_*lo*_ represents the sleep-promoting neurons in the five layers of Figure 1, the control signal provided via Con_lo_ corresponds to five firing rates being selected from the set Σ_*lo*_ and their resultant induction of an element in the set Q_lo_. The high-level system in Figure 3 will also be defined via a four-tuple

(6) $$\begin{array}{r} G_{hi}=\left(\Sigma_{hi},\;Q_{hi},\;\delta_{hi},\;q_{hi,0}\right) \end{array}$$

with

(7) $$\begin{array}{l} \Sigma_{hi}=\left\{w_{1}^{+},w_{1}^{-},\;w_{2}^{+},\;w_{2}^{-},\;\ldots ,\;w_{5}^{+},\;w_{5}^{-}\right\}\\ Q_{hi}=\left\{{\text{wake}}_{1},{\text{wake}}_{2},\;\ldots ,{\text{wake}}_{\text{K}}\right\} \end{array}$$

The events associated with the high-level controller denote the activity of the wake-promoting neurons of the five layers either exceeding (+ superscript) or not-exceeding (– superscript) a threshold associated with wake (w). The control signal provided via the Con_hi_ channel represents the five firing rates selected from the set Σ_*hi*_ inducing an element from the set Q_hi_. With decreasing subscript values corresponding to a higher valence toward wakefulness, the induction of state wake_1_ may correspond to the activity of all five populations of wake-promoting neurons implicating wakefulness. The statements with respect to the transition function δ and the initial state q_0_ for the low-level control system also apply to the high-level control system. While it is expected that δ_*lo*_ ≠ δ_*hi*_ and *q*_*lo*.0_ ≠ *q*_*hi*,0_, similar obstacles exist so far as determining the transition function and an initial state for the high-level system.

In considering the DES of Figure 3 as a model for the regulation of sleep, the presence of sensory input will be accounted for through the activity of the wake and sleep promoting neural populations. The information communicated by the channels Inf_lo_ and Inf_hi_ may encompass two sets of messaging necessary for the sleep and wake promoting neurons, respectively, to adapt their activity. The presence of a command channel Com_hilo_ and the absence of reciprocal Com_lohi_ channel accounts for the wake-promoting neurons' extensive projections to wake as well as sleep centers, while the sleep-promoting neurons do not have such extensive projections. Rather, the sleep-promoting neurons only project locally to other sleep centers via Con_lo_ and do not have the same potency in the induction of the behavioral state. The cross-level information channel Inf_lohi_ may account for the aggregate effect of the sleep center on arousal, although this effect is dwarfed by overall arousal. Indeed, the model does not contain a reciprocal Inf_hilo_ channel because the wake-promoting system's hierarchical superiority to the sleep-promoting system obviates the need for G_hi_ to directly provide information to G_lo_, rather the system C_hi_ provides commands to C_lo_.

With the possibility of the discussed theory and hypothesis guiding future experiments, it is natural to ponder if the effects of pharmacology or lesions can be reflected in the controllers C_lo_ and C_hi_ as well as the states of the G_lo_ and G_hi_ systems. Such considerations would shed light on the causal relationship between brain regions and the REM, NREM, and wake phenotypes. Unfortunately, it is difficult to claim that the mathematical operations within the four blocks in Figure 3 are presently known at the degree necessary to allow for such analysis.

In our proposed model, arousal neurons (C_hi_) dominate sleep-promoting neurons (C_lo_). Our model predicts that simultaneous stimulation of sleep-promoting and wake-promoting neurons will result in wakefulness. Strong sensory inputs driving wake-promoting neurons like PB can maintain wakefulness (Cano et al., 2008). Similarly, high sleep pressure and artificial stimulation of wake-promoting neurons lead to wakefulness, followed by sleep pressure, despite evidence of increased activity in sleep-active neurons in the PZ (Qiu et al., 2016). This model also predicts that simultaneous inhibition or lesion of arousal and paired sleep neurons would increase sleep, mimicking the loss of the dominant, wake-promoting neurons.

## Model Predictions and Hypotheses

The two presented models assume that a degree of hierarchy exists between the neural circuits that govern sleep as well as among those that govern arousal. The presence of such hierarchy as well as the communication among the various nodes are a crucial aspect of the models discussed above. The layered model would predict several aspects of sleep-wake dynamics that are testable with current technology. For instance, the presented models imply that caudal sleep-wake centers have a stronger overall effect on an animal's behavioral state. Using an optogenetic approach, we would predict that stimulating sleep-promoting terminals in caudal regions, like the PZ (inhibiting the PB) would have a stronger sleep-promoting effect than stimulating terminals from the VLPO in the LH. We would also expect that stimulating local terminals (PZ terminals in the PB) would have stronger effects than more distant terminals since PZ projects to more rostral sleep-promoting centers. Similarly, the model would predict that stimulation of caudal wake-promoting regions such as the PB will produce increased wakefulness in comparison to the stimulation of more rostral wake-promoting regions.

Another prediction of our model is that every rostrocaudal layer of the brain will contain sleep-active and wake-active neurons. Because sleep-active and wake-active neurons are intermingled in the BF and cortex, dissecting out specific populations may require opto- or chemogenetic or other approaches (Anaclet et al., 2015, 2018; Xu et al., 2015). Future research will likely uncover novel sleep and wake-promoting neuronal groups. Cortical sleep-promoting regions, for example, may be found via retrograde tracing from wake-promoting regions and confirmed by methods in functional circuit tracing (Agostinelli et al., 2017). The presented model hypothesizes that newly discovered sleep-promoting groups will have strong but neuroanatomically local projections to wake-promoting neurons, while wake-promoting groups will project more widely and to other wake-promoting regions.

In the presented model the dominance of sleep-wake control is progressively reduced from the brainstem to the hypothalamus, basal forebrain, basal ganglia, and finally to the cerebral cortex. Arousal nodes at the aforementioned brain regions connect to each other and innervate the entire CNS in order to promote wakefulness, while the nodes implicated in causing sleep inhibit the arousal system nodes at the same layer in the architecture. Arousal nodes, regulated by sensory, circadian, and homeostatic inputs, dominate by driving sleep and wake behavior. Conversely, sleep systems can suppress local wake-promoting nodes, but the overall sleep state requires a reduction in the activity of arousal nodes as well. Two models that draw upon control system theory have been discussed as representing the dynamics associated with the induction of arousal. First, a series of computations were presented for an architecture based on Brooks' subsumption model where controller, system, and sensor pairs cascade to provide an arousal signal. Secondly, a DES model has been suggested as capturing the hierarchical interaction, command, and information exchange that is necessary to attain an aggregate state from a series of underlying processes. While providing differing levels of abstraction and analytical rigor, both models incorporate layering among their components and provide a degree of hierarchy between the layers. The two models also highlight the need to consider feedback and coordination among the neural networks implicated in sleep and arousal. Our novel algorithmic approach considers sleep-wake features such as slow sleep onset, quick wake onset, and stabilized sleep-wake state. The model may drive a behavioral interpretation of sleep, wakefulness, and arousal, while also providing testable hypotheses within the sleep-wake system of different mammalian species using novel techniques. While it is evident that convincing the veracity of the presented models will require additional data and ensuing causal analysis, we have discussed means by which the models, experiments, and analysis can abet one-another to provide an understanding of a neurological function.

## Author Contributions

MC, SS, J-SL, and JL wrote the manuscript.

### Conflict of Interest

All funding sources supporting this work are fully acknowledged. MC was employed by PureTech Health. The remaining authors declare that the research was conducted in the absence of any commercial or financial relationships that could be construed as a potential conflict of interest.
